# GD2-specific chimeric antigen receptor-modified T cells for the treatment of refractory and/or recurrent neuroblastoma in pediatric patients

**DOI:** 10.1007/s00432-021-03839-5

**Published:** 2021-11-01

**Authors:** Lihua Yu, Lulu Huang, Danna Lin, Xiaorong Lai, Li Wu, Xu Liao, Jiale Liu, Yinghua Zeng, Lichan Liang, Guanmei Zhang, Bin Wang, Zhu Wu, Shaohua Tao, Yuchen Liu, Cheng Jiao, Lung-Ji Chang, Lihua Yang

**Affiliations:** 1 Department of Pediatric Hematology, Zhujiang Hospital, Southern Medical University, Guangzhou, Guangdong China; 2 Department of Neonatology, Zhujiang Hospital, Southern Medical University, Guangzhou, Guangdong China; 3 Department of Pediatric Intensive Medicine, Zhujiang Hospital, Southern Medical University, Guangzhou, Guangdong China; 4 Shenzhen Geno-Immune Medical Institute, Shenzhen, China

**Keywords:** Neuroblastoma, CAR T, Immunotherapy, Pediatric, GD2

## Abstract

**Purpose:**

This study aimed to evaluate the safety and efficacy of chimeric antigen receptor (CAR) disialoganglioside 2 (GD2)-specific (4SCAR-GD2) T cells for treatment of refractory and/or recurrent neuroblastoma (NB) in pediatric patients.

**Experimental design:**

A phase I clinical study using 4SCAR-GD2 T cells for the treatment of NB in pediatric patients was conducted. This study was registered at www.clinicaltrials.gov (NCT02765243). A lentiviral CAR with the signaling domains of CD28/4-1BB/CD3ζ-iCasp9 was transduced into activated T cells. The response to 4SCAR-GD2 T-cell treatment, and 4SCAR-GD2 T-cell expansion and persistence in patients were evaluated. Toxicities were determined based on the National Cancer Institute Common Terminology Criteria for Adverse Events (CTCAE) v4.03.

**Results:**

Twelve patients were enrolled and finally ten patients were included in this clinical trial which started from January 1, 2016, to August 1, 2017. These patients had progressive disease (PD) before CAR T-cell infusion. After 4SCAR-GD2 T-cell treatment, 6 (6/10) had stable disease (SD) at 6 months, and 4 (4/10) remained SD at 1 year and alive after 3–4 years of follow-up. Six patients died due to disease progression by the end of July 1, 2020. The median overall survival (OS) time was 25 months (95% CI, 0.00–59.43), and the median progression-free survival (PFS) time was 8 months (95% CI, 0.25–15.75). Grade 3 or 4 hematological toxicities were the common adverse events frequently occurred after fludarabine and cyclophosphamide (Flu/cy) chemotherapy. Grade 1–2 toxicities such as cytokine release syndrome (CRS) and neuropathic pain were common, but were transient and mild.

**Conclusions:**

The 4SCAR-GD2 T-cell therapy demonstrated antitumor effect and manageable toxicities, indicating its potential to benefit children with refractory and/or recurrent NB.

**Supplementary Information:**

The online version contains supplementary material available at 10.1007/s00432-021-03839-5.

## Introduction

Neuroblastoma (NB) is the most prevalent extracranial pediatric solid tumor occurring typically in young children (Sherief et al. 2019). Patients with refractory and/or recurrent NB have a poor prognosis, even after intensive treatment with chemotherapy, surgery, stem cell transplantation, radiation, and/or antibody-based therapy (Mallepalli et al. 2019). Although metaiodobenzylguanidine (MIBG) and disialoganglioside 2 (GD2) monoclonal antibody treatment might be effective, they are difficult to access, resulting in worse prognosis of NB in China (Su et al. 2020).

Disialoganglioside 2 (GD2) is a surface antigen expressed on most neuroectoderm-derived cells including NB, melanoma, and others. GD2 is also expressed less predominantly in normal human tissue cells, such as neurons, skin melanocytes, and peripheral sensory nerve fibers (Ho et al. 2016). GD2 has been an effective target for NB immunotherapy and anti-GD2 monoclonal antibodies (MoAbs) based on the 3F8 and CH14.18 clones have been approved for clinical application (Yu et al. 2010; Kushner et al. 2011). The frequent and severe pain resulting from anti-GD2 antibody therapy is the main adverse event limiting dose escalation.

Chimeric antigen receptor (CAR) T cells, as a new therapeutic paradigm, have demonstrated remarkable antileukemic activity against B cell hematological malignancies (Part et al. 2018; Maude et al. 2018). However, their efficacy against solid tumors has not yet been clearly illustrated. In a previous study, the first- generation GD2 CAR T cells comprised of EBV- cytotoxic T lymphocytes (CTLs) and conventionally activated T cells showed no neurological toxicity, and some patients with active NB even achieved complete response (CR) (Pule et al. 2008; Louis et al. 2011). The third-generation GD2 CAR T cells had similar antitumor effects with significant expansion but the effect was transient. Furthermore, their effects were not greater than those earlier studies of the first-generation GD2 CAR T cells and the first-generation GD2-CAR EBVST (Heczey et al. 2017). The latest research, a second-generation GD2-CAR with CD28/CD3 signaling domain incorporating a humanized anti-GD2 single-chain variable fragment (scFv) based on the K666 antibody, revealed excellent safety and on-target off-tumor toxicity in patients, which illustrated transient antitumor effects (Straathof et al. 2020).

Here we developed a new generation of GD2-CAR T cells (4SCAR-GD2) to evaluate their safety and efficacy for refractory and recurrent NB treatment. The 4SCAR-GD2 used autologous T cells transduced with a lentiviral vector to express the anti-GD2 antibody domain scFv with a CD3-zeta domain, a 4-1BB (CD137) domain, a CD28 extracellular and intracellular domains and an inducible caspase 9 domain. In the 4SCAR-GD2 design, we devised a suicide gene, inducible caspase 9, into the vector construct, without impairing the antitumor efficacy of the CAR T cells. Accumulated studies showed that such design could prevent severe adverse effects (Budde et al. 2013, Gargett and Brown 2014a). Similar research-based an extended CD28 costimulatory signal plus a CD27 signaling domain with an inducible caspase 9 suicide gene, with target-specificity for CD19 surface antigen, has successfully applied in Chinese patients with relapsed or refractory B cell leukemia and achieved CR in up to 70–80% of patients (Xu et al. 2020; Zhang et al. 2018a).

## Methods

### Study design and participants

The study was approved by the Institutional Review Board of Zhujiang Hospital, affiliated with Southern Medical University (2016-EKZX-001). The study was registered at www.clinicaltrials.gov (NCT02765243). The protocol was conducted according to the principles of the Declaration of Helsinki. Written informed consents were provided by the guardians of all patients.

This was a single-cohort, phase I study of 4SCAR-GD2 T cells in children with refractory and/or recurrent NB. Patients received fludarabine and cyclophosphamide (Flu/cy) for lymphodepletion (Cy at 300 mg/m^2^/dose on days -4, -3, and-2 and Flu at 25 mg/m^2^/dose on days -4, -3, and -2 prior to 4SCAR-GD2 T-cell administration). The participants received once or more an intravenous infusion of modified 4SCAR-GD2 T cells and were closely followed-up for treatment-related responses. Additional treatment was not administrated after 4SCAR-GD2 T-cell treatment in patients without progression.

When the 4SCAR-GD2 T cells were not detectable in the peripheral blood in the follow-up, patients might opt to receive additional 4SCAR GD2 T-cell infusion. The additional CAR T-cell products were prepared independently using the cryo-preserved peripheral mononuclear cells or re-collection of PBMC from the patients.

### Assessment of clinical response and toxic effects

The response to 4SCAR-GD2 T-cell infusion was evaluated by computed tomography (CT), positron emission tomography (PET) or magnetic resonance imaging (MRI) on the primary tumor sites, and a bone scan and bone marrow aspirate and biopsy were performed to evaluate metastatic sites after the initial infusion, according to the international neuroblastoma response criteria (INRC) (Tu et al. 2019). Further imaging was conducted again at 6 and 12 months after infusion. The response assessment results were recorded at 6 and 12 months after infusion. Overall survival (OS) and progression-free survival (PFS) were determined for evaluating the clinical response to 4SCAR-GD2 cells treatment. The patients had not received ^123^I‐MIBG scans as the latter was not easily accessible in China. Efficacy parameters included the following: stable disease (SD), partial response (PR) or progressive disease (PD); complete remission (CR); PFS (time from first treatment to disease progression) and OS. Toxicities were assessed through physical examinations, performance tests and laboratory tests of organ function weekly in the first month, each month in 6 months and at the end of the study. Adverse events were graded based on the National Cancer Institute (NCI) Common Terminology Criteria for Adverse Events (CTCAE) v4.03 (https://evs.nci.nih.gov/ftp1/CTCAE/CTCAE_4.03_2010-06-14_QuickReference_5x7.pdf), and cytokine release syndrome(CRS) was graded according to a revised grading system (Park et al. 2018a).

### GD2 expression by immunohistochemical (IHC) staining

Formalin-fixed paraffin-embedded tumor sections were immunohistochemically stained with mouse anti-human GD2 antibody (BD Biosciences, Cat# 554,272, 0.5 mg/mL). The isotype control was stained with purified mouse IgG2a (BioLegend, Cat# 400,202). All images were captured from tumor sections using a Zeiss Axio Vert.A1 microscope and Zeiss ZEN imaging software. The tissue staining intensity was compared with that from positive and negative controls and scored from 0 to 4 according to two components: staining intensity and the percentage of positive cells. Each sample was assessed and graded by two independent observers.

### Construction of the 4SCAR-GD2 lentiviral vector

**Fig. 1:**
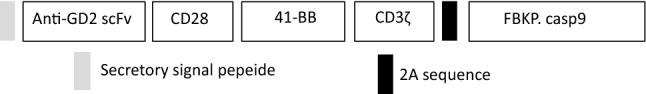
4SCAR-GD2 T cells design: the GGGGS-linker was included between the signaling domains, and the 2A linker was included in between the CD3z and iCasp9 domains

Lentiviral vectors were generated using the NHP/TYF lentiviral vector (LV) system as previously described (Lee et al. 2014; Chang et al. 2005). The DNA sequences of the anti-GD2 CAR clone hu3F8(a humanized mouse 3F8 scFv) were optimized for human codons, chemically synthesized, cloned into the lentiviral vector pTYF and packaged into lentiviral particles for gene transfer (Wang et al. 2006; Ahmed et al. 2014). The final lentiviral constructs were verified by restriction enzyme mapping and DNA sequencing. The GD2 CAR sequences were constructed with a lentiviral CAR design (4SCAR). The 4SCAR incorporated several intracellular T cells signaling motifs including the CD28 extracellular/transmembrane and cytoplasmic domains, the costimulatory 4-1BB intracellular TRAF-binding domain, the CD3ζ chain intracellular domain, and an inducible cell death-initiating caspase 9 genetic cassette (4SCAR-GD2) (Fig. 1). The iCasp9 system has been published in previous studies, and the small molecule dimerizer drug AP1903 could induce CAR T cells apoptosis (Cheung et al. 2012).

### T-cell isolation and 4SCAR-GD2 T-cell production

Peripheral blood mononuclear cells (PBMCs) of patients were collected by apheresis and blood buffy coats were prepared. PBMCs were isolated using Ficoll-Paque plus (GE Healthcare). T cells were activated using anti-CD3(MACS CD3 MicroBeads 130-097-043) antibody-conjugated magnetic beads and anti-CD28 (Invitrogen anti-Hu CD28 clone 28.2 16-0289-85) antibody. T cells were maintained in AIM-V (Invitrogen, Thermo Fisher Scientific, Waltham, MA) supplemented with interleukin (IL)-2, IL-7 and IL-15, as previously described (Gargett and Brown 2014b). Phenotypic analysis of the activated cells was performed to confirm T-cell purity. After expansion for two-days, T cells were transduced with the lentiviral 4SCAR-GD2 vector and quality control/assurance assay for CAR T killing function was routinely performed as an integrated program. The 4SCAR-GD2 T cells were collected and infused five days after transduction. Transduction efficiency is reported as the percentage of CAR DNA copies positive per cellular genomes based on a standard single-cell clone tumor cell line with one copy of transgene per cell, and the internal house-keeping gene GAPDH; for example, 100% means 1 copy of CAR per T cell. Safety tests included mycoplasma detection and endotoxin analysis which were conducted and passed before CAR T-cell infusion.

### Immunophenotyping of T-cell products

The following anti-human antibodies were used to evaluate the phenotype of the cells: anti-hCD3 PE (MACS, 130,113,129), anti-hCD4 Pacific Blue (BD Biosciences, 558,116), anti-hCD8 PerCp-Cy5.5 (BD Biosciences, 565,310), and anti-hCD8 PE-Cy7 (BD Biosciences, 557,747). Data acquisition was performed using a CytoFLEX flow cytometer (Beckman).

### CAR detection by real-time quantitative PCR (qPCR)

The CAR copy number in the blood was determined by real-time qPCR, based on both SYBR and TaqMan probe methods, as previously described (Okada et al. 2015; Cui et al. 2003; Nair et al. 2019; Zhang et al. 2018b). Genomic DNA was harvested from blood cells using a Promega Genomic DNA Purification Kit (Promega Corp, Madison, WI, USA). The qPCR data were collected using MX3000P (Stratagene, Agilent Technologies, Santa Clara, CA, USA) after 4SCAR-GD2 T-cell treatment at 0, 1, 2, and 3 weeks and each month until the CAR copy number was at undetected level.

### Cytokine analysis based on a cytokine bead array (CBA)

The serum levels of the cytokines and cytokine receptors TNF-α, IL-2R, IL-1β, IL-8, IL-6 and IL-10 were determined by using the BD CBA Human Soluble Protein Flex Set System at 0, 1, 2, 3 weeks and so on (Arican et al. 2005). The CBA system captures soluble analytes with beads of a known size and fluorescence and allows quantification using flow cytometry according to the manufacturer’s manual.

### Statistical analysis

Descriptive statistics (means, ranges and standard deviations or standard errors) were used to summarize data. Correlation analysis was performed using Spearman’s rank correlation coefficient. OS and PFS were estimated by the Kaplan–Meier method. A two-sided *p* value < 0.05 was considered statistically significant. Analyses were performed with GraphPad Prism 6 (GraphPad Software) and IBM SPSS Statistics for Windows, version 21.0 (IBM Corp., Armonk, NY, USA).

## Results

### Patient characteristics

**Fig. 2:**
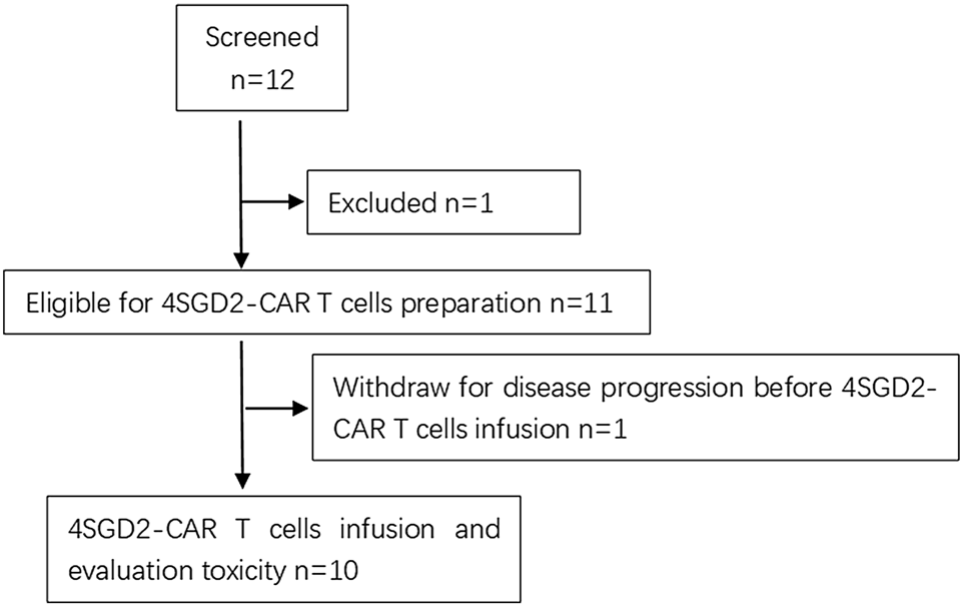
Recruitment and treatment on study

From January 1, 2016, to August 1, 2017, a total of 12 patients were screened, and 10 patients with relapsed (2/10) or refractory (8/10) disease were enrolled in the study (Fig. 2). Seven of the 10 patients were male with a median age of 4.5 years (range 1.8–7 years). The previous treatments of the patients were chemotherapy, surgery, radiation and/or stem cell transplantation (Table S1 in the Supplementary Appendix). All patients were treated with 4SCAR-GD2 T cells (Table 1). Three patients had unresectable tumors at the primary site 1 in the spinal canal and 2 in the abdomen), three patients had bone marrow diseases, and the remaining three patients had multiple bone lesions, one of whom had lymph node involvement at the time of infusion. In the follow-up, when the 4SCAR-GD2 T cells could no longer be detected in the blood of patients No.1, 2 and 7, the guardians/families consented to additional CAR T-cell infusions. As a result, patient No.1 received 4SCAR-GD2 T cells for three times at an interval of 3 months, while patients No.2 and 7 received 4SCAR-GD2 T cells for two times at intervals of 6 months and 3 months, respectively.

**Table 1:** Clinical characteristics of the NB patients enrolled in this study

No.	Age (years)	Sex	Histological category^a^	Stage at initiation	Status before CART infusion	Disease burden at enrollment^b^	Immunohistochemistry	CAR T cells × 10^6^(per/kg)^c^	CAR LV transduction efficiency (%)	Peak CAR DNA copies (%)
1	5	F	GNB (MYCN^−^)	4	Relapsed	Bone, Bone marrow	GD2 3 +	0.41	15.78	0.02
								3.05	88.97	0.02
								0.43	7.53	0.05
2	6	M	NB (MYCN^−^)	4	Refractory	Bone	GD2 2 +	0.8	34.00	2.27
								34	596.08^d^	1.48
3	6	F	NB (MYCN^−^)	4	Refractory	Bone	GD2 3 +	0.2	5.33	7.93
4	6	M	NB (MYCN^−^)	4	Refractory	Bone, Bone marrow	GD2 3 +	1.81	68.00	2.69
5	1.7	M	NB (MYCN^+^)	4	Refractory	Residual soft tissue	GD2 2 +	1.4	39.06	15.42
6	2	M	NB (MYCN^−^)	3	Refractory	Residual mass tumor	GD2 3 +	1.3	24.42	5.03
7	4	F	NB (MYCN^−^)	4	Relapsed	Bone marrow	GD2 2 +	5.05	88.68	3.98
								6.34	98.16	0.06
8	2	M	GNB (MYCN^−^)	3	Refractory	Residual soft tissue	GD2 2 +	1.81	29.76	6.38
9	2	M	NB (MYCN^+^)	4	Refractory	Bone	GD2 2–3 +	0.13	3.59	12.83
10	6	M	NB (MYCN^−)^	4	Refractory	Lymph node	GD2 2–3 +	4.19	97.72	22.4

*NB* neuroblastoma; *GNB* ganglioneuroblastoma

^a^The MYCN oncogene status is given in parentheses. MYCN + indicates amplified MYCN; MYCN − indicates non-amplification

^b^Disease was evaluated before CAR-T-cell infusion

^c^Dose of CAR  T cells infusion

^d^In CAR T cell preparation, 500% means 5 copies of lentiviral vector genes inserted in one T cell

### Clinical response and survival assessments

Patient No.1 and 7 had NB bone marrow relapse, and CAR T cells expanded poorly after infusion of 4SCAR-GD2 T cells. Their CAR T-cell counts in the peripheral blood were lower than detection sensitivity. Therefore, they received multiple infusions but developed bone marrow relapse at the end.

Patients No. 2, 3, 4, and 9 had multiple skeletal metastatic lesions but negative in bone marrow. The efficacy of 4SCAR T cells treatment was assessed by observing survival because ^123^I‐MIBG scans evaluation was not available. Intracranial metastases occurred in both patients No.3 and 9. Patient No.3 had a posterior cranial fossa mass and received total surgical resection, but developed skeletal recurrence 1.5 years later. Patient No. 9 had intracranial metastases combined with intratumoral hemorrhage and died of brain herniation. Patients No. 2 and 4 were long-term survivors.

Patient No.6 had a huge abdominal occupancy with residual 50 × 42 × 23 mm on abdominal CT. The residual tumor was 54 × 33 × 22 mm on six months and one year later after 4SCAR-GD2 T-cell infusion. No significant abnormality in the radiological distribution of the residual tumor was seen on PET-CT. Patient No.8 had a spinal cord occupancy that could only be partially resected by surgery, and the residual tumor was slightly smaller than before after 4SCAR-GD2 T cells therapy. Both patients were long-term survivors.

Patient No.5 and 10 had NB soft tissue residuals and developed distal site recurrence after a period of remission after 4SCAR-GD2 T treatment.

**Fig. 3:**
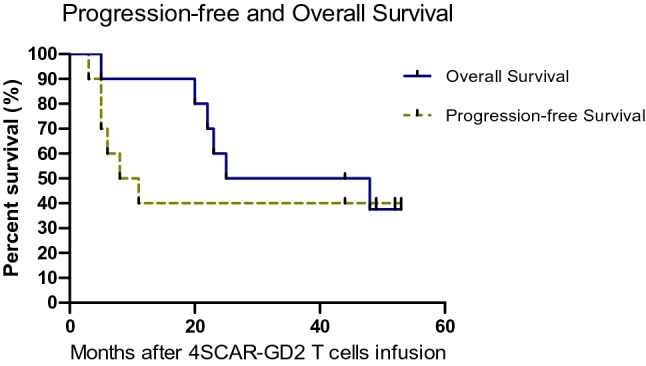
Long-term survival of patients who received 4SCAR-GD2 T cell therapy (PFS and OS of all the enrolled patients are shown)

The clinical responses to 4SCAR-GD2 T-cell therapy are summarized in Table 2. Of the 10 patients who had PD before CAR T-cell infusion, 6 achieved SD at 6 months, and the remaining 4 patients had SD at 1 year and alive at least 3–4 years of follow-up. The median overall survival (OS) time was 25 months (95% CI, 0.00 to 59.43), and the median progression-free (PFS) survival time was 8 months (95% CI 0.25–15.75). Six patients who relapsed again after 4SCAR-GD2 T-cell infusion received salvage chemotherapy, but these patients died due to disease progression. The survival curves for all patients are shown in Fig. 3.

**Table 2:** Response and prognosis after 4SCAR-GD2 T-cell therapy

No.	Response at 6 months	Response at 1 year	Disease re-progression (months)^a^	Re-relapsed sites	Time of 4SCAR-GD2 T cell last detected (days after infusion)	Time to follow-up (years)	Long-term outcome
1	SD	PD	11	Bone marrow	34	2	DOD
2	SD	SD	–	–	148	4	SD
3	PD	PD	3	Cerebral metastasis	114	4	DOD
4	SD	SD	–	–	57	4	SD
5	PD	PD	5	Bone and soft tissue masses	121	1	DOD
6	SD	SD	–	–	102	4	SD
7	PD	PD	8	Bone marrow	72	1	DOD
8	SD	SD	–	–	510	3	SD
9	PD	–	5	Cerebral metastasis	158	0	DOD
10	SD	PD	6^a^	Soft tissue mass	189	1	DOD

^a^Patient 10 relapsed after the sixth month of evaluation

*PD* progressive disease; *CR* complete response; *PR* partial response; *SD* stable disease; *DOD* died of the disease

### Characterization, expansion, and persistence of 4SCAR-GD2 T cells

**Fig. 4:**
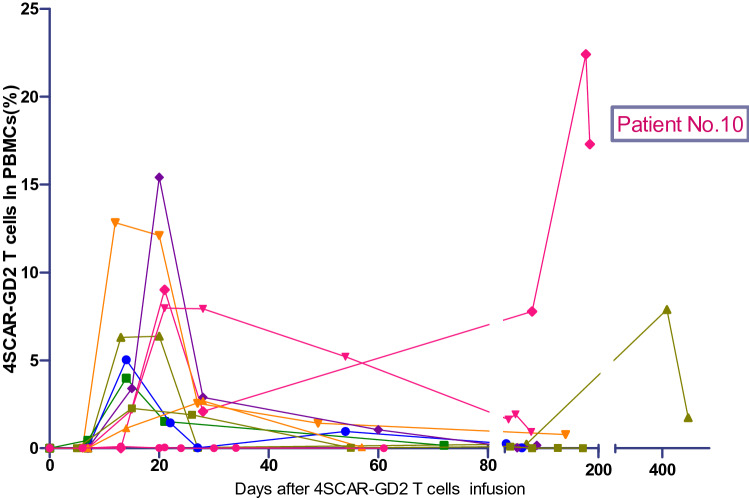
Kinetics of 4SCAR-GD2 T cells in the peripheral blood (The percentage (%) of 4SCAR-GD2 T cells in peripheral blood mononuclear cells was assessed by qPCR before and after 4SCAR-GD2 infusion in ten enrolled patients). The 4SCAR-GD2 T cells expanded significantly when the disease relapsed in patient No.10 after six months

The dose of infused CAR T cells ranged from 0.13–34 × 10^6^/kg body weight. The median lentiviral vector gene transfer efficiency was 36.53%, ranging from 3.59% to 596.08% (500% means 5 copies of lentiviral vector genes inserted in one cell). Cell products contained both CD4^+^ and CD8^+^ T cells (median ratio CD4:CD8 = 0.81; range, 0.15–4.27) (Table S2 in the Supplementary Appendix). The 4SCAR-GD2 T cells were released for infusion after a median preparation time of 7–8 days. The CAR DNA copies were measured to be 0.02%–22.4% in the 10 patients (Fig. 4). The number of 4SCAR-GD2 T cells in patient No.10 was markedly expanded when the disease re-relapsed after six months. Moreover, the 4SCAR-GD2 T cells in patient No.8 with 1.74% CAR DNA copies were detected in the blood for more than 1 year (in the 17th month).

### Safety assessment

Toxicities were mostly self-limited and resolved soon after treatment with the 4SCAR-GD2 T cells. There was no central neurotoxicity, and intervention or supportive care was not necessary (Table 3). No toxic event-related death was observed in this trial. The common treatment-related toxicities after Flu/cy chemotherapy conditioning were hematological events (neutropenia and thrombocytopenia) (Table S3 in the Supplementary Appendix). These events were fully reversible without any treatment in all patients within a median of 4 days (range, 0–22 days). Acute capillary leak syndrome (4/10) and CRS (9/10) of grades 1–2 were observed approximately 1 week after infusion. A febrile reaction (9/10) was the first and most common symptom observed. Fever without infection and febrile neutropenia occurred within a median of 10 days after infusion (range 0–18 days) and lasted for a mean of 5.9 days (range 0–9 days). The highest temperature was 40 °C and lasted more than 24 h in four patients after 4SCAR-GD2 T-cell infusion at the end of the first week. Hypotension (1/10) and neuropathic pain (3/10) were rare, transient, and mild in the 4SCAR-GD2 T-cell-treated patients. Cough (6/10) and acneiform rash (4/10) were common symptoms (median, 7 days; range, 5–20 days). Eosinophilia was observed in all patients in the phase of CAR T-cell expansion. There were no late-onset toxicities or long-term adverse events in the 4SCAR-GD2 T-cell-treated patients during follow-up.

**Table 3:** Toxic effects after 4SCAR-GD2 T-cell infusion

Toxic Effect	Grade				
	1	2	3	4	Total
CRS	4	5			9
CRES					0
Fever without infection	3	2	4		9
Hypotension	1				1
Neuropathic pain	3				3
Cough	3	3			6
Rash acneiform	2	1	1		4
Acute capillary leak syndrome		4			4
Neutropenia		1	3	5	9
Thrombocytopenia	2		1	2	5

*CRES* CAR T-cell-related encephalopathy syndrome; *CRS* Cytokine release syndrome

**Fig. 5:**
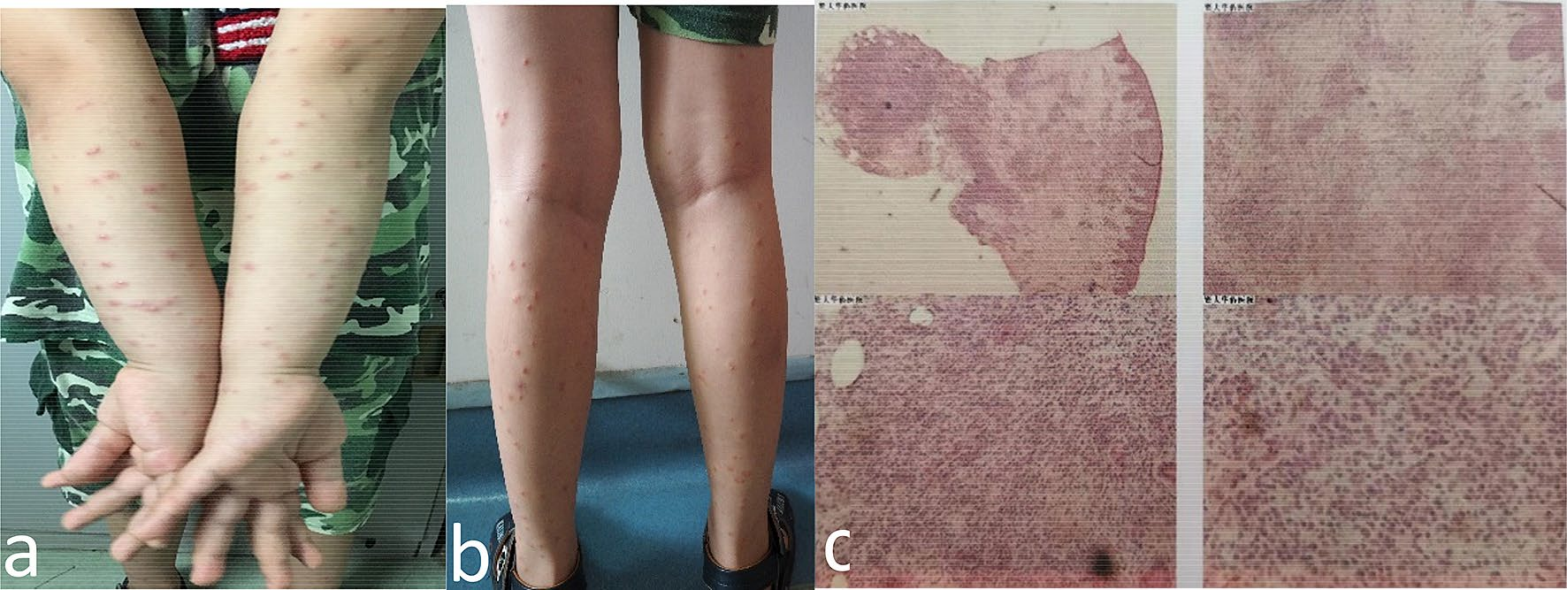
Extensive papules on the face, scalp, and limbs (**a** and **b**). The skin biopsy: hyperkeratosis with parakeratosis of epidermis, hyperacanthosis and a great number of lymphocytes around the perivascular base of superficial middle dermis, which were the mainly inflammatory cell infiltration (**c**)

The patient No.10 experienced extensive papules on the face, scalp, and extremities, that were accompanied with symptoms of pruritus and tenderness with deflorescence over one month. The symptoms receded without any treatment (Fig. 5). Pathological biopsy suggested immune-mediated reaction.

**Fig. 6:**
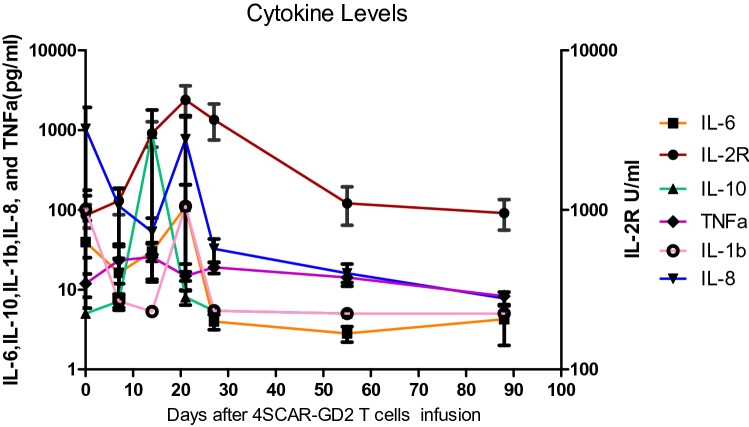
Serum cytokine kinetics after 4SCAR-GD2 T-cell therapy (Data are presented as the Mean ± Sem)

The cytokine profiles after 4SCAR-GD2 T-cell infusions were similar in all cases, with high secretion of IL-2R, IL-10, TNF-α, IL-8, IL-6 and IL-1β (Fig. 6). The serum levels of the cytokines were elevated when fever developed. The highest level of ferritin, IL-6 and IL-10 were 3417 ug/L, 1000 pg/ml and 8014 pg/ml, respectively (Table S3). There were no correlations between CAR T-cell expansion and serum cytokine levels, except for the level of IL-2R (*r* = 0.857; *p* = 0.014) (Fig. S1).

## Discussion

In China, patients with refractory and/or recurrent NB have a poor prognosis in the late stages (Arican et al. 2005). This study first assessed GD2 CAR T-cell therapy on the basis of costimulatory signals with CD28-4-1BB plus an inducible domain caspase 9 in a lentiviral vector system. The results showed that 4SCAR-GD2 T cells were safe in children with relapsed/refractory NB. Previous studies were based on the first-, second- and third-generation CAR T cells with both CD28 and OX40 costimulatory domains (Pule et al. 2008; Louis et al. 2011; Heczey et al. 2017). Preclinical research indicated that a 3rd-generation CAR incorporating the CD28 and 4-1BB costimulatory domains improved antitumor efficacy compared with the combination of CD28.OX40 domains (Quintarelli et al. 2018). In addition, persistence or exhaustion of T cells may be the main factors contributing to the antitumor effectiveness of GD2 CAR T-cell therapies. CAR T-cell engineering with a combination of multiple signaling domains has been used to improve the safety and efficiency of CAR T cells and in vivo persistence (Jafarzadeh et al. 2020). Previous research showed that high-affinity GD2-specific E101K CAR T cells enhanced antitumor efficacy as compared with the m3F8 CAR T cells in vivo, yet it was concomitantly associated with lethal neurotoxicity more severe than the m3F8 CAR design (Richman et al. 2018, Richman and Milone 2018). Because a murine version of the scFv of the GD2 antibody could result in exhaustion of T cells in vivo thus limit antitumoral efficacy, we incorporated a humanized GD2 scFv antibody moiety in the 4SCAR-GD2 CAR design, which effectively suppressed tumor growth and prolonged patients’ survival without neurotoxicity as reported by others previously (Straathof et al. 2020).

It has been demonstrated that Flu/cy lymphodepleting chemotherapy could enhance CAR T-cell expansion and promote persistence (Louis et al. 2011; Heczey et al. 2017). In this study, 4SCAR-GD2 T-cell persistence was demonstrated to last longer than 6 months after infusion. Due to the limited number of patients, the correlation between the infusion dose and CAR T-cell expansion was not found, and there was no apparent correlation between CAR T-cell expansion and patient response. There was a correlation between early disease progression after CAR T-cell infusion and a poor prognosis. Furthermore, the patients with metastatic bone marrow disease and N-MYC amplification had the worst responses. The patient No.8 who had 1.74% CAR DNA copies detected in the blood for more than 1 year, experienced the longest survival time after the 3 years of follow-up suggesting that the increased 4SCAR-GD2 T-cell persistence could result in prolonged survival.

Previous clinical studies support that the 4SCAR design has a high safety profile and mild cytotoxicity (Xu et al. 2020; Tu et al. 2019). None of the patients in this study required the agent AP1903 to eliminate the CAR T cells because immediate severe toxicity at the time of administration and long-term toxicity were not observed.

CRS is the most common concern in CAR T-cell therapy, especially for the CD19-specific CAR T-cell therapy in patients with leukemia. Application of the anti-IL-6 receptor antibody tocilizumab is a routine management protocol (Titov et al. 2018; Mueller et al. 2018). In this study, CRS was moderate and no severe acute adverse effects were recorded. Although positive correlations between CRS, and tumor burden or CAR T-cell expansion in vivo have been reported in other CAR T-cell studies (Gardner et al. 2017), no such correlation was found in our study.

Toxicity of the hematopoietic system was reported after CAR T-cell therapy, especially after CD19 CAR T-cells therapy (Fried et al. 2019). Some researchers believe that this toxicity is attributed mostly to the lymphodepleting chemotherapy regimen and CRS, characterized by a biphasic effect (Nahas et al. 2020; Schaefer et al. 2019). In our study, cytopenia occurred after the lymphodepleting chemotherapy and also occurred lightly in the late period due to moderate CRS.

It is interesting that the 4SCAR-GD2 T cells markedly expanded when the disease re-relapsed (patient No. 10) after six months, suggesting that re-recurrence of the tumor could stimulate the proliferation of 4SCAR-GD2 T cells again. This phenomenon supports the existence of memory 4SCAR-GD2 T cells in patients, but the rate of tumor growth might be faster than CAR T-cell expansion. The high relapse rate after 4SCAR-GD2 T-cell therapy remains a major challenge. Many factors contribute to a high relapse rate, such as tumor immune escape mechanisms, the immunosuppressive tumor microenvironment, perturbation of antigen presentation, and metabolic alterations in tumor cells, to name a few (Vanichapol et al. 2018).

Because no relationship between infusion dose and T-cell expansion was observed, optimal dose of CAR T cells treatment is uncertain (Maude et al. 2018). The tumor relapse rate might be decreased by several approaches, such as optimizing CAR density, affinity and sensing, improving immunological synapse formation, and combining treatment with oncolytic viruses or PD-1 inhibitor in the future trials (Watanabe et al. 2018).

In conclusion, 4SCAR-GD2 T-cell therapy for relapsed and/or refractory NB patients delayed tumor progression with little to no toxicities, suggesting that 4SCAR-GD2 CAR T-cell treatment could be a safe and efficient approach for the management of NB.

## Supplementary Information

Below is the link to the electronic supplementary material.

Supplementary file1 (DOCX 61 kb)

## Data Availability

Available upon request.
