# Supplementary material for: GD2-specific chimeric antigen receptor-modified T cells for the treatment of refractory and/or recurrent neuroblastoma in pediatric patients
Source: J Cancer Res Clin Oncol. 2021 Nov 1;148(10):2643–52. doi: 10.1007/s00432-021-03839-5 (PMC9470713; doi:10.1007/s00432-021-03839-5)
Supplement: Supplementary file 1 — Supplementary file1 (DOCX 61 kb) [file 432_2021_3839_MOESM1_ESM.docx]

Supplementary Information

**GD2-specific Chimeric Antigen Receptor-modified T Cells for the Treatment of**

**Refractory and/or Recurrent Neuroblastoma in Pediatric Patients**

Lihua Yu^1†^, Lulu Huang^1†^, Danna Lin^1^, Xiaorong Lai^1^, Li Wu^1^, Xu Liao^1^, Jiale Liu^1^, Yinghua Zeng^1^, Lichan Liang^1^, Guanmei Zhang^1^ Bin Wang^1^, Zhu Wu^1^, Shaohua Tao^1^, Yuchen Liu^2^, Cheng Jiao^2^, Lung-Ji Chang^2*^, Lihua Yang^1*^

Table S1. Clinical characteristics of the NB patients before enrolled in this study

| No. | Age at Dx  (years) | Previous therapy* | | | |
| --- | --- | --- | --- | --- | --- |
|  |  | Surgery | Chemotherapy  (cycle) | XRT | Auto-HSCT |
| 1 | 3 | GTR | 20 | Y | N |
| 2 | 5 | GTR | 8 | Y | N |
| 3 | 5 | GTR | 8 | Y | N |
| 4 | 4 | STR | 16 | Y | N |
| 5 | 1.2 | STR | 8 | N | N |
| 6 | 1 | STR | 10 | Y | N |
| 7 | 3 | GTR | 7 | Y | Y |
| 8 | 1.3 | STR | 10 | Y | N |
| 9 | 1.7 | GTR | 10 | Y | Y |
| 10 | 5 | STR | 11 | Y | N |
| *MIBG and GD2 monoclonal antibody treatment are not accessible in China. Abbreviations: NB, neuroblastoma; Dx, diagnosis; XRT, radiation therapy; GTR, gross total resection; STR, subtotal resection. | | | | | |

Table S2. T Cell products phenotype

| No. | phenotype (%) | | | | CD4:CD8  ratio |
| --- | --- | --- | --- | --- | --- |
|  | CD3^+^ | CD4^+^ | CD8^+^ | CD56^+^ |  |
| 1 | 6 | 2 | 3 | 1 | 0.67 |
|  | 82 | 21 | 54 | 15 | 0.39 |
|  | 98 | 59 | 35 | 1 | 1.69 |
| 2 | 78 | 9 | 62 | 18 | 0.15 |
|  | 96 | 44 | 43 | 5 | 1.02 |
| 3 | 90 | 41 | 46 | 13 | 0.89 |
| 4 | 58 | 9 | 42 | 34 | 0.21 |
| 5 | 90 | 27 | 57 | 12 | 0.47 |
| 6 | 91 | 39 | 53 | 9 | 0.73 |
| 7 | 97 | 53 | 37 | 1 | 1.43 |
|  | 100 | 67 | 33 | 1 | 2.03 |
| 8 | 98 | 57 | 33 | 5 | 1.72 |
| 9 | 96 | 64 | 15 | 5 | 4.27 |
| 10 | 98 | 34 | 51 | 9 | 0.67 |

Table S3. 4SCAR-GD2 T cells hematologic toxicities, ferritin, IL-6 and IL-10

| No. | Hematologic toxicities | | Ferritin  (ug/L) | | IL-6  (pg/mL) | | IL-10  (pg/mL) | |
| --- | --- | --- | --- | --- | --- | --- | --- | --- |
|  | Neutrophil count (× 109/liter) | Platelet count (× 109/liter) |  |  |  |  |  |  |
|  | Min | Min | Min | Max | Min | Max | Min | Max |
| 1 | 0.17 | 11 | 853.3 | 1108 | 2.7 | 3.5 | <5 | - |
|  | 0.52 | 114 | ND | ND | <2 | - | <5 | - |
|  | 0.34 | 114 | ND | ND | ND | ND | ND | ND |
| 2 | 1.1 | 134 | 18.4 | 56.6 | <2 | 11 | <5 | 6.79 |
|  | 1.45 | 149 | ND | ND | ND | ND | ND | ND |
| 3 | 0.49 | 70 | 695.8 | 1348 | <2 | 8.08 | <5 | 15.7 |
| 4 | 0.51 | 97 | 1300 | 1538 | <2 | 10.4 | <5 | 11.9 |
| 5 | 0.19 | 90 | 535.9 | 1534 | <2 | 52.7 | <5 | 34.3 |
| 6 | 1.49 | 136 | 434.8 | 802 | 2.19 | 58.4 | <5 | 13.1 |
| 7 | 0.48 | 170 | 534.4 | 887.2 | 7.99 | 60.7 | 5.35 | 8.63 |
|  | 0.87 | 211 | 455.4 | 574.7 | ND | ND | ND | ND |
| 8 | 0.72 | 124 | 789.5 | 2149 | <2 | 72.3 | <5 | 17.9 |
| 9 | 0.26 | 11 | 1480 | 3417 | 3.13 | 34.4 | <5 | 11.6 |
| 10 | 0.86 | 147 | 137.5 | 330.3 | 4.12 | 1000 | <5 | 8014 |
| ND, Not detected. | | | | | | | | |


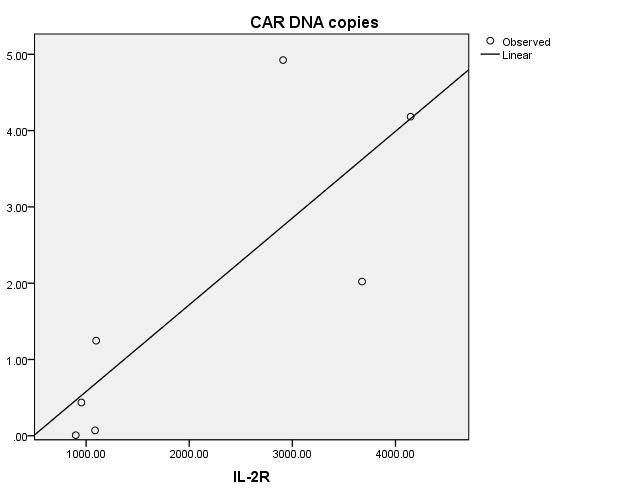


**Fig. S1** The correlation between 4SGD2-CAR T cells expansion and IL-2R
